# Cathepsin K deficiency prevented stress-related thrombosis in a mouse FeCl_3_ model

**DOI:** 10.1007/s00018-024-05240-0

**Published:** 2024-05-04

**Authors:** Xueying Jin, Xueling Yue, Zhe Huang, Xiangkun Meng, Shengnan Xu, Yuna Wu, Ying Wan, Aiko Inoue, Megumi Narisawa, Lina Hu, Guo-Ping Shi, Hiroyuki Umegaki, Toyoaki Murohara, Yanna Lei, Masafumi Kuzuya, Xian Wu Cheng

**Affiliations:** 1 Department of Cardiology and Hypertension, Jilin Provincial Key Laboratory of Stress and Cardiovascular Disease, Yanbian University Hospital, 1327 Juzijie, Yanji, 133000 Jilin People’s Republic of China; 2 Department of Community Health Care and Geriatrics, Nagoya University Graduate School of Medicine, Nagoya, Aichi 466-8550 Japan; 3 Department of Neurology, University of Occupational and Environmental Health, Kitakyushu, Fukuoka 807-8555 Japan; 4 Department of Vascular Surgery, The Second Affiliated Hospital, Zhejiang University School of Medicine, Hangzhou, 310000 Zhejiang People’s Republic of China; 5 Institute of Nano-Life-Systems, Institutes of Innovation for Future Society, Nagoya University Institute of Innovation for Future Society, Nagoya University, Nagoya, Aichi-Ken 466-8550 Japan; 6 Department of Cardiology, Nagoya University Graduate School of Medicine, Nagoya, Aichi 466-8550 Japan; 7 Department of Public Health, Guilin Medical College, Guilin, 541199 Guangxi People’s Republic of China; 8 Department of Medicine, Brigham and Women’s Hospital and Harvard Medical School, Boston, MA 02115 USA; 9 Meitetsu Hospital, Nagoya, Aichi 451-8511 Japan; 10 Key Laboratory of Natural Medicines of the Changbai Mountain, Ministry of Education, Yanbian University, Yanji, 133002 Jilin People’s Republic of China; 11 Department of Intensive Care, Yanbian University Hospital, 1327 Juzijie, Yanji, 133000 Jilin People’s Republic of China

**Keywords:** Chronic stress, Endothelial injury, Cathepsin K, Thrombosis, Apoptosis

## Abstract

**Background:**

Exposure to chronic psychological stress (CPS) is a risk factor for thrombotic cardiocerebrovascular diseases (CCVDs). The expression and activity of the cysteine cathepsin K (CTSK) are upregulated in stressed cardiovascular tissues, and we investigated whether CTSK is involved in chronic stress-related thrombosis, focusing on stress serum-induced endothelial apoptosis.

**Methods and results:**

Eight-week-old wild-type male mice (CTSK^+/+^) randomly divided to non-stress and 3-week restraint stress groups received a left carotid artery iron chloride3 (FeCl_3_)-induced thrombosis injury for biological and morphological evaluations at specific timepoints. On day 21 post-stress/injury, the stress had enhanced the arterial thrombi weights and lengths, in addition to harmful alterations of plasma ADAMTS13, von Willebrand factor, and plasminogen activation inhibitor-1, plus injured-artery endothelial loss and CTSK protein/mRNA expression. The stressed CTSK^+/+^ mice had increased levels of injured arterial cleaved Notch1, Hes1, cleaved caspase8, matrix metalloproteinase-9/-2, angiotensin type 1 receptor, galactin3, p16^IN4A^, p22phox, gp91^phox^, intracellular adhesion molecule-1, TNF-α, MCP-1, and TLR-4 proteins and/or genes. Pharmacological and genetic inhibitions of CTSK ameliorated the stress-induced thrombus formation and the observed molecular and morphological changes. In cultured HUVECs, CTSK overexpression and silencing respectively increased and mitigated stressed-serum- and H_2_O_2_-induced apoptosis associated with apoptosis-related protein changes. Recombinant human CTSK degraded γ-secretase substrate in a dose-dependent manor and activated Notch1 and Hes1 expression upregulation.

**Conclusions:**

CTSK appeared to contribute to stress-related thrombosis in mice subjected to FeCl_3_ stress, possibly via the modulation of vascular inflammation, oxidative production and apoptosis, suggesting that CTSK could be an effective therapeutic target for CPS-related thrombotic events in patients with CCVDs.

**Supplementary Information:**

The online version contains supplementary material available at 10.1007/s00018-024-05240-0.

## Introduction

Chronic psychological stress (CPS) in modern lifestyles has been shown to be closely associated with the incidence of many diseases including multiple sclerosis, neurodegeneration, cancer, lung thrombosis, muscle atrophy, and diabetes mellitus [1–4]. Exposure to CPS is also a comparatively intractable risk factor for thrombotic cardiocerebrovascular diseases (TCVDs) [5–9]. A 2023 review highlighted the close relationship between chronic stress and the prothrombotic state and thrombus formation in animal models and humans with TCVDs [10].

There are common consequences of the pathophysiological events that occur in response to harmful obesity-related adipose changes and various psychological stressors, including immune and inflammatory cell infiltration, insulin resistance, and the prothrombotic state [4]. For example, atherosclerosis-prone apolipoprotein E (ApoE)-deficient (ApoE^−/−^) mice were subjected to variable chronic stress, and the mice then exhibited activated hematopoiesis and the promotion of atherosclerotic lesion features that are associated with vulnerable plaques that trigger stroke and acute myocardial infarction in humans [11, 12]. It was also reported that in rats and mice subjected to 2 weeks of restraint stress, an elevated level of dipeptidyl peptidase 4 (DPP-4) stimulated the proliferation of bone marrow hematopoietic stem cells and their release into peripheral blood through a β-adrenergic receptor-3/CXCL12-mediated mechanism that is dependent on glucagon-like peptide-1 and its receptor axis [13]. One of our studies also demonstrated that in mice subjected to immobilized stress, elevated plasma DPP-4 promoted the thrombosis that had been induced by iron (III) chloride (FeCl_3_) via a negative modulation of plasma and/or vascular ADAMTS13 (a disintegrin-link and metalloproteinase with thrombospondin type 13 motifs) and oxidative stress production and inflammation [14]. Stress can produce a thromboembolism, causing thrombotic events in both animal models and humans with coronary and cerebral arterial atherosclerotic lesions [5–8]. It is thus essential to explore how stressors affect the homeostasis of the fibrinolysis and coagulation systems in humans with TCVDs.

The expressions of members of the cathepsin family are upregulated during various forms of cardiovascular and muscle diseases [15–17]. Some cathepsins are located from the lysosomes to other cellular spaces and have exhibited non-traditional functions [18–21]. Recent evidence suggests that the cysteine cathepsin K (CTSK) plays a significant role in insulin receptor substrate (IRS)-1 ubquitination in cachexia-related muscle atrophy [18]. In mice, cathepsin S (CTSS) activity controlled vascular remodeling and experimental hyperplasia via the modulation of p38 mitogen-activated protein kinase (MAPK) signaling and an Akt-histone deacetylase (HDAC)-6 signaling pathway in response to injuries [22]. Among the cathepsin family members, CTSK has been shown to modulate vascular cell apoptosis by the modulation of caspase-8 maturation under pathological conditions in vivo and in vitro [23]. We also demonstrated that in a murine model, CTSK played an important role in proteinuria, hypertension, and renal remodeling in response to stress and/or a 5/6 nephrectomy, possibly via reductions of glomerular fibrosis, inflammation, and apoptosis [24]. Another of our group’s studies revealed that chronically stressed atherosclerotic plaques had increased CTSK but also a relatively reduced expression of the endogenous inhibitor cystatin C [25], indicating a shift in the imbalance between the endogenous inhibitors and cathepsins that leads to vascular endothelial damage and remodeling under CPS.

Here, we explored the role(s) of CTSK in the pathogenesis of chronic stress-related thrombosis in mice subjected to chronic restraint stress conditions. Stress-related carotid artery endothelial injury and apoptosis and thrombus formation were examined in wild-type (CTSK^+/+^) mice and CTSK-knockout (CTSK^−/−^) mice that were subjected to chronic stress. In a separate in vivo experiment, CTSK^+/+^ mice underwent stress conditions and were then treated with or without a specific CTSK inhibitor (CTSK-II). By conducting in vitro experiments, we investigated CTSK-mediated Notch1 activation and its downstream apoptotic signaling pathway in human vein umbilical endothelial cells (HUVECs) in genetic (gene silencing and overexpression approaches) and pharmacological (specific and non-specific CTSK inhibitor) interventions of CTSK and a γ-secretase activity assay. The results demonstrated that the silencing and the overexpression of CTSK in HUVECs respectively increased and reduced cleaved Notch1 [c-Notch1/Hes1 and cleaved caspase-8 (c-Casp8)] levels in response to stressed serum and oxidative stress. We also observed that a pharmacological inhibition of CTSK suppressed stress serum-induced c-Notch1 production in HUVECs and that recombinant human CTSK (rhCTSK) degraded a γ-secretase substrate in a dose-dependent manner, whereas rhCTSS, rhCTSB, and rhCTSV had no effects. These findings might constitute the first evidence and mechanistic explanation of the CTSK-dependent activation of Notch1/Hes1-caspase-8 signaling in arterial endothelial cell apoptosis and thrombus formation under our experimental stress conditions. We propose that CTSK is an important molecular determinant of thrombotic events and a potential therapeutic target in patients with CCVDs.

## Materials and methods

### Antibodies and reagents

The commercially available antibodies used in all of the experiments were as follows: anti-p38MAPK (cat. no. 144511), anti-phospho-p38 mitogen-activated protein kinase (p-p38MAPK; cat. no. 4511), anti-Notch1, and anti-c-Notch1 were purchased from Cell Signaling Technology (Danvers, MA, USA). Anti-AT1R (sc-1173) and anti-glyceraldehyde 3-phosphate dehydrogenase (GAPDH; cat. no. sc-20357) were purchased from Santa Cruz Biotechnology (Santa Cruz, CA). Anti-gp91^phox^ (clone: 53/gp91^phox^) was from BD Biosciences (Bedford, MA). Anti-CD31 (ab28364) and anti-galectin-3 (ab76245) were from Abcam (Cambridge, MA). Fluorescein isothiocyanate (FITC)-labeled goat anti-rabbit IgG (cat no. K1203) was purchased from APExBio (Houston, TA). Anti-p16-INK4A (cat. no. 10883-1-AP) and anti-homolog-1 (Hes1) were purchased from Proteintech (Chicago, IL). Antibody of p16 (cat. no. 10883-1-AP) was from Proteintech (Rosemont, IL). Antibody of CD31 (#550274) was purchased from BD Pharmingen (San Diego, CA). Rabbit pAb (ab28364) and FITC goat anti-mouse IgG (cat no. K1203) were from APExBio.

The enzyme-linked immunosorbent assay (ELISA) kits for the vascular cell adhesion molecule-1 (VCAM-1; MVC00) was from R&D Systems (Minneapolis, MN), and ADAMTS13 (cat. no. CSB-EL001301MO) was purchased from Cusabio (Houston, TX). Plasminogen activator inhibitor-1 (PAI-1, #ab197752) and von Willebrand factor (vWF, #ab208980) were purchased from Abcam. The SYBR™ Green Master Mix and RNeasy Micro Kits were purchased from Qiagen (Hilden, Germany). Nontargeting control short interfering (si)RNA (#F5129292-921) and CTSK-specific siRNA (siCTSKs: #F5129442005, #F5129442006) were purchased from Sigma-Aldrich (St. Louis, MO). The RNeasy Micro Kits and SYBRTM Green Master Mix were from Qiagen. CTSK plasmid was from Invitrogen (Carlsbad, CA). The ImmPACT™ DAB peroxidase substrate (cat. no. SK-4105) was from Vector Laboratories (Burlingame, CA). The DCTM protein assay kit (cat. no. 500-0114) was purchased from Bio-Rad Laboratories (Hercules, CA). The SuperScript III First Strand and Lipofectamine® 3000 Transfection Kits were purchased from Invitrogen. The Amersham ECL Prime Western Blotting Detection kit was from GE Healthcare (Freiburg, Germany). The nitrocellulose transfer membrane was from Amersham Bioscience (Piscataway, NJ). The 0.5 w/v% sterilized methyl cellulose 400 solution was purchased from FujiFilm Wako Pure Chemical Corp. (Osaka, Japan). The In-Fusion HD cloning kit was purchased from Takara Bio (#639648; Kusatsu, Shiga), and the CEQ8000 Genetic Analyzer was purchased from Beckman Coulter (Brea, CA). The HUVECs were purchased from Cell Applications (San Diego, CA). Endothelial growth medium (EGM)-2 SingleQuots and endothelial basal medium (EBM)-2 were from Lonza (Walkersville, MD). The In Situ Cell Death Detection Kit (TUNEL: terminal deoxynucleotidyl transferase dUTP nick end labeling) was purchased from Roche Diagnostics (Lot#10131400, Mannheim, Germany).

CTSK-II (also known as a CTSK specific inhibitor; [1-(N-Benzyloxycarbonyl-leucyl)-5-(N-Boc-phenylalanyl-leucyl)carbohydrazide] (also known as CTSL inhibitor V (called CTSL-V); [Z-Phe-Tyr(O*t*Bu)-COCHO·H_2_O], CA-074Me (also known as CTSB inhibitor IV); [l-3-*trans*-(propylcarbamoyl)oxirane-2-carbonyl]-l-isoleucyl-l-proline methyl ester), E-64d (also known as non-specific cathepsin inhibitor); [(l-3-*trans*-ethoxycarbonyloxirane-2-carbonyl)-l-leucine (3-methylbutyl) amide], E64 (also known as a non-specific cathepsin inhibitor); N-[N-(L-3-Trans-carboxirane-2-carbonyl)-L-leucyl]-agmatine), and GM6001 (also known as an MMP inhibitor) were purchased from Calbiochem (San Diego, CA). Fluorescence-quenching substrate (also known as γ-secretase substrate; Nme-Asp-Gly-Cys-Gly-Val-Leu-Leu-Lys-DNP-Arg-Arg-NH2), recombinant human MMP-1 (rhMMP-1), and recombinant human membrane type 1 of MMP (rhMT1-MMP) were purchased from Enzo Life Sciences (Farmingdale, NY). DAPT (also known as the γ-secretase inhibitor IX); (3,5-difluorophenylacetyl)-l-alanyl-l-2-phenylglycine *t*-butyl ester] and L-685,458 (also known as γ-secretase inhibitor X); [(2*R*,4*R*,5*S*)-2-benzyl-5-(*t*-butyloxycarbonylamino)-4-hydroxy-6-phenylhexanoyl]-l-leucyl-l-phenylalanine amide) were purchased from the Peptide Institute (Osaka, Japan). Recombinant human CTSB (rhCTSB), rhCTSK, rhCTSV, and rhCTSS were purchased from R&D Systems. Anti-fluorescence quenching sealing solution with DAPI for the visualization of the cell nuclei was purchased from Beyotime (Shanghai, China). All powdered chemical inhibitors were dissolved in sterilized dimethyl sulfoxide (DMSO) and added to the cell culture medium to yield 0.2% DMSO as a final concentration.

### Animal care and use

Seven-week-old male wild-type mice (CTSK^+/+^, C57BL/6J background; Chubu Kagaku Shizai, Nagoya, Japan) and CTSK-knockout mice (CTSK^−/−^ [26], C57BL/6 J background; a gift from Harvard University, Boston, MA) were provided tap water *libitum* and a standard diet for 1 week. All animals were kept in a facility maintained at 22 °C room temperature with a 12-h dark/light cycle. The mice used in the experiments weighed between 22 and 26 g. The animal study protocols were approved by the Institutional Animal Care and Use Committees of Nagoya University (Protocol nos. 30121 and 30068 for the experiments with CTSK^+/+^ and CTSK^−/−^ mice) and Yanbian University Hospital (Protocol no. 2021106 for the wild-type mice and cellular experiments) and were performed in accord with the Guide for the Care and Use of Laboratory Animals published by the U.S. National Institutes of Health. The animals were treated by trained research staff in accord with the guidelines of the Institutional Animal Care and Use Committees of Nagoya University and Yanbin University.

### Restraint stress procedure

For the examination of the effects of chronic restraint stress on thrombus formation, we first randomly divided the 8-week-old mice into a control group (the non-stress group) and an immobilization stress group (the restraint stress group; *n* = 39 for each group) that was subjected to the immobilization described below for 3 weeks. All of the mice were then subjected to the left carotid artery thrombus-induction surgery described below prior to morphological and biological analyses. To prevent the animals from becoming accustomed to the immobilization stress, we combined three types of stress over each week from Monday to Sunday, and we altered the stress order [11]. The details of the three types of stress are given in Supplementary Table S1 [25].

### Mouse carotid artery thrombosis model and treatments

The mouse carotid artery iron chloride3 (FeCl_3_)-induced thrombosis model was made as described [14]. Briefly, the mouse was anesthetized with a xylazine (1 mg/mL)-ketamine (10 mg/mL) cocktail (0.01 mL/g body weight). After the left carotid artery was exposed, it was tied off and wrapped with filter paper that had been saturated with 20% FeCl_3_ solution. The filter paper (3 mm) was left in place for 15 min and then removed. When the color of the artery wall had darkened, we ligated both ends of the filter paper strip with suture thread according to the length of the filter paper strip in order to sample the left carotid artery accurately. The mice in the non-stress group underwent a sham operation (without thrombosis induction) plus the isolation of control arteries. The length and weight of the wet thrombi were evaluated by a trace scale and measuring tape. Blood was isolated from the left ventricle of each mouse for biological analyses with an ELISA.

### CTSK deletion and inhibition experiments

For the evaluation of CTSK deletion-mediated protection against thrombus formation, we randomly assigned CTSK^+/+^ and CTSK^−/−^ mice to four groups: two non-stress groups (N-CTSK^+/+^ and N-CTSK^−/−^ groups, *n* = 38 for each group) and two stress groups (S-CTSK^+/+^ and S-CTSK^−/−^ groups, *n* = 38, each group). All four groups of mice were then subjected to the carotid artery thrombosis model described above. In separate CTSK inhibition experiments, CTSK^+/+^ mice were randomly divided into two groups and loaded with either a vehicle (0.5% carboxymethylcellulose, by oral gavage; Stress) or the specific CTSK inhibitor (CTSK-I, 5 mg/kg; *n* = 38 each group) once daily for 3 weeks under daily 4-h restraint stress, and then subjected to the left carotid artery thrombus-induction surgery.

### Sample collection and the evaluation of thrombus weight and length

After the restraint stress regimen and the thrombus induction with and without pharmacological treatment, the mice were sacrificed by an intraperitoneal overdose of sodium pentobarbital (50 mg/kg) and then perfused with phosphate-buffered saline (PBS) at physiological pressure, and the left carotid artery was sampled. For the histological examinations, the carotid artery was embedded in OCT after fixation with 4% paraformaldehyde for 16 h (4 °C). For the biological analyses, the carotid arteries were kept in liquid nitrogen (for the protein assay) and RNAlater™ solution (Thermo Fisher Scientific, Waltham, MA) (for the gene assay). The weights of the inguinal adipose tissues were measured, and the tissues were maintained in RNAlater solution for the targeted gene evaluations.

### Quantitative real-time gene expression assay

Total RNA was isolated from the tissues and cells and tissues with the RNAeasy Mini Kit as described [27]. The mRNA was reverse-transcribed to cDNA with a Superscript III first-strand synthesis system for a quantitative polymerase chain reaction (qPCR) assay of the following targeted genes: CTSS, CTSK, CTSL, gp91^phox^, p22^phox^, monocyte chemoattractant protein-1 (MCP-1), intracellular adhesion molecule-1 (ICAM-1), tumor necrosis factor-alpha (TNF-α), interleukin-1beta (IL-1β), MMP-9, and MMP-2. The primer sequences of the targeted genes are provided in Supplementary Table S2. Each targeted gene’s expression was normalized to the related internal GAPDH gene.

### Western blot analysis

Proteins were isolated from the tissues and cells using lysis buffer (1% Triton X-100, 20 mM Tris–Cl, 150 mM NaCl, 0.05% SDS, 1 mM EDTA, 1% Na-deoxycholate, and fresh 1 × protease inhibitors; pH 8.0). Following the measurement of protein concentrations by the DC Protein Assay kit (Bio-Rad), the proteins were equally loaded and separated by sodium dodecyl sulfate–polyacrylamide gel electrophoresis (SDS-PAGE) and then transferred to FluoroTrans-W® membranes (Cytiva, Marlborough, MA) for treatment overnight with the first antibodies (1:1,000 for each antibody). The membranes were then incubated with the secondary horseradish peroxidase-conjugated antibody (1:10,000–15,000). Protein levels evaluated from the western blots were normalized by GAPDH.

### Morphometry and immunohistochemistry analyses

Cross-cryosections (4 μm) of the vessels were made and then stained with hematoxylin and eosin (H&E) for the histological evaluation. For the characterization of the endothelial injury/loss, we applied immunostaining using anti-CD31 to corresponding sections of the arterial tissues at day 21 after the stress and thrombosis induction. After being washed twice with PBS, the tissue sections were incubated with mouse IgG (1:200) for 1 h, and the results were visualized with an M.O.M. substrate kit (Vector Laboratories) [14]. We evaluated images of sections immunostained for CD31^+^ with the use of ImagePro software (BZ9000 Analysis, Keyence, Osaka, Japan). Cross-sections (*n* = 5–7) of each vessel were examined and averaged for each mouse. The data are presented as the CD31-positive cell numbers of the fixed endothelial area (0.5 mm^2^) that included lesions.

### Immunofluorescence assay

The immunofluorescence assay was performed as described [27]. Following blocking with bovine serum albumin for 30 min, the carotid artery sections were treated with a rabbit polyclonal antibody to CD31 (1:100) overnight. After being washed with PBS for three times, the tissue sections were treated with the secondary antibody against anti-rabbit IgG for 1 h at room temperature, and then the nuclei were counterstained with an anti-fluorescence quenching solution including DAPI. Sections were imaged using an EVOS FL Auto 2 imaging system (Thermo Fisher Scientific).

### Transmission electron microscopy

Transmission electron microscopy (TEM) was performed as described [28]. Mouse aortic tissue was cut into approx. 1-mm^3^ pieces and fixed for 24 h with 0.16 M PBS containing 2% glutaraldehyde (pH 7.2) and then for 1 h with 1% osmium tetroxide. Following dehydration in a graded series of ethanol solutions, the fixed aortic tissues were exposed to propylene oxide and embedding in Epon. The sections were cut (60–70 nm) and then stained with uranyl acetate and lead citrate. A transmission electron microscope was used to visualize and evaluate vessel micro-morphological changes, especially endothelial injury and loss, at magnifications of 6000× and 10,000× [24]. Sections (*n* = 7–8) were quantified for each mouse, and histograms were created separately for each mouse group.

### ELISAs and biochemical analyses

For the ELISAs, at the indicated time points, blood was isolated directly from the left ventricles of the mice. After centrifugation at 1000 g for 10 min, the supernatants of the blood samples were separated. The plasma was measured with mouse vWF, PAI-1, VCAM-1, and ADAMTS13 ELISA kits according to the manufacturers’ protocols.

### Cell cultures

HUVECs were cultured in EGM-2 at 37 °C with a humidified atmosphere of 95% air and 5% CO_2_. The cells were seeded in six-well (4 × 10^5^ cells/well) plates in EBM-2 for 6 h and treated in the presence or absence of H_2_O_2_ (0, 200, or 400 μmol/L) in EBM-2 for 24 h. The results were applied to the cellular assays. For special experiments, the non-stress serum and stressed serum were collected from the non-stressed mice and stressed (loaded stress for 10 days) mice.

### The silencing and the overexpression of CTSS

CTSK knockdown was performed as described [26]. Briefly, cells were grown on 60-mm dishes until 80% confluence. Following treatment with non-targeting control siRNA, silamin A/C (positive control siRNA), and siCTSK (final siRNA concentration of 100 pM for each), respectively, for 48 h, the cells were subjected to PCR and western blotting assays. Transfected cells were also used for cellular functional experiments [18].

For the overexpression assay, CTSK plasmid was transformed in competent *E*. *coli* cells by using the heatshock method, followed by purification using a Qiagen plasmid mini-kit [24]. HUVECS were transfected by CTSK plasmid (pl-CTSK) with the help of Lipofectamine LTX & Plus reagents (Thermo Fisher Scientific).

### TUNEL staining

The HUVECs transfected by siCTSK or pl-CTSK were incubated in serum-free Dulbecco’s modified Eagle’s medium (DMEM) containing 400 µM H_2_O_2_ for 24 h and then subjected to TUNEL staining. HUVECs were also cultured with non-stress or stressed serum for 24 h and then subjected to a TUNEL staining assay [27].

### Assay for γ-secretase activity

The assay for γ-secretase activity was performed as described [26]. Briefly, the extracts of the HUVECs were incubated with 8 μmol/L of an intramolecularly quenched fluorogenic peptide probe, harboring the substrate for γ-secretase in buffer containing 2.5 mM EDTA, 50 mM sodium acetate, 1 mM DTT, and 0.01% Triton X-100 (pH 6.8) for 24 h. Twenty micromoles per liter of the sham fluorogenic peptide probe was incubated with recombinant human CTSB (rhCTSB), rhCTSK, rhCTSL, or rhCTSS, or with rhMT1-MMP or rhMMP-1 in the presence or absence of the related protease inhibitors at their respective optimal pH conditions. Fluorescence was measured using a plate reader (Fluoroskan™ Labsystems Ascent CF, Thermo Fisher Scientific) with an excitation wavelength of 355 and an emission wavelength of 440 nm.

### Statistical analyses

Data are expressed as the mean ± standard error of the mean (SEM). The Shapiro–Wilk test was used to determine the normality of data distribution. The Kruskal–Wallis test was applied for data sets for which normal distribution could not be assumed. Student’s *t-*tests (for two-group comparisons) or a one-way analysis of variance (ANOVA) (three or more-group comparisons) followed by Tukey’s post hoc tests were used for the statistical analyses. The body weight (BW) data were subjected to a two-way repeated-measures ANOVA and Bonferroni post hoc tests.

For sample sizes of <5/group, two-group comparisons were performed by the Mann–Whitney rank-sum test, and multigroup comparisons were performed by a Kruskal–Wallis one-way ANOVA with Tukey’s post hoc test. The sample number (*n*) per experimental group is noted in the figure legends and experiment-specific notes. All morphological analyses were evaluated by two observers in a blinded manner, and the values they obtained were averaged by two other observers. Statistical analyses were performed using GraphPad Prism ver. 9.3.1 (GraphPad, La Jolla, CA). A probability (*p*)-value < 0.05 was considered significant.

## Results

### Stress accelerated arterial endothelial damage and thrombus formation

**Fig. 1:**
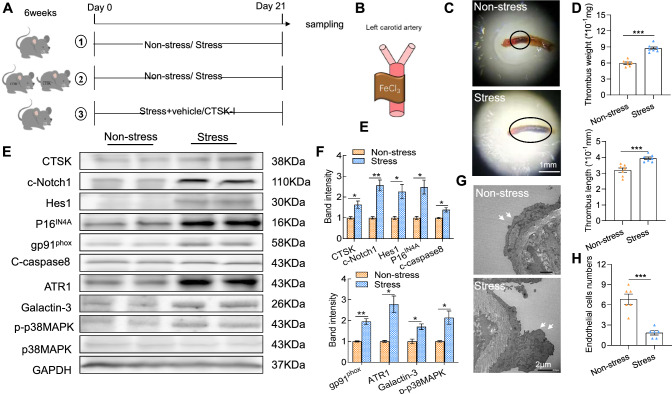
The stress protocol stimulated the arterial expression of CTSK protein and accelerated FeCl_3_-indcued carotid artery thrombus formation. **A** Schematic diagram of the carotid artery thrombus operation and sampling procedures in mice under the immobilized stress conditions. **B** Photographs of FeCl_3_-treated carotid arteries from mice with and without stress. **C**, **D** Representative photos and quantitative data exhibiting the length and weight of the thrombi in the two groups (*n* = 6–7). **E**, **F** Representative western blotting images and quantitative data showing the level of CTSK protein in both groups (*n* = 3 each). **G**, **H** Representative transmission electron microscopy (TEM) images and the quantitative data presenting the numbers of endothelial cells of the injured arteries in the two experimental groups (*n* = 6 each). Data are mean ± SEM. *p < 0.05, **p < 0.01, ***p < 0.001 vs. the control group by unpaired Student’s *t*-test. *Scale bars* 1 mm for the photographs and 2 μm for the TEM

For the investigation of the impact of chronic stress on artery thrombosis, we subjected CTSK^+/+^ mice to the FeCl_3_ injury combined with a sham operation or stress. Figure 1A is a schematic diagram of the FeCl_3_-induced artery thrombosis induction and sampling procedure at the suggested timepoints, and Fig. 1B provides photographs of FeCl3-treated carotid arteries from mice with and without stress. The stressed mice exhibited decreased inguinal adipose volumes and body weights (Suppl. Fig. S1A–C). These observations showed that the stress markedly enhanced both the arterial thrombus weights and the size of the thrombi (Fig. 1C, D). The quantitative thrombus-area assay of the H&E staining yielded the same conclusions (Suppl. Fig. S1D), suggesting that chronic stress accelerates arterial thrombus formation in response to FeCl_3_ induction.

### Stress stimulated CTSK expression, Notch1 signaling activation, oxidative stress production, inflammation, and apoptosis

To study potential mechanisms of chronic stress-related thrombosis, we assessed the levels of CTSK and the Notch1 (c-Notch1 and Hes1)-, senescence (p16^IN4A^)-, apoptosis (c-caspase8 and p-p38MAPK)-, oxidative stress (gp91^phox^)-, and inflammation (ATR1 and galactin-3)-related proteins of the injured/stressed arteries. As shown in Fig. 1E, F, the stressed vessel tissues had markedly elevated levels of these investigated molecules compared to the non-stressed arteries, suggesting that there was a close relationship between the thrombus formation and CTSK overexpression accompanied by harmful changes in c-Notch1, apoptosis-, inflammation-, and oxidative stress-related molecules in the carotid arteries in response to chronic stress + FeCl_3_ induction.

The quantitative data obtained in the TEM and the immunostaining assays conducted as a next step to evaluate the endothelial cells’ quality and integrity revealed a dramatic loss of endothelial cells in the lesions of the stressed vessel tissues (Fig. 1G, H, Suppl. Fig. S1E). The chronic stress markedly increased the expressions of both proteolytic enzyme-related genes (CTSK, CTSS, MMP-2, and MMP-9) and inflammation-related genes (TLR-4, MCP-1, ICAM-1, and TNF-α) compared to the non-stressed CTSK+/+ mice (Suppl. Fig. S1F–I). The ELISA data revealed that the levels of vVW, VCAM1, MCP-1, PAI-1, and IL-18 were elevated in the stressed mice, and the levels of plasma ADAMTS13 were decreased in those of the non-stressed mice (Suppl. Fig. S2).

### CTSK deletion ameliorated apoptosis, oxidative stress production, and inflammation

**Fig. 2:**
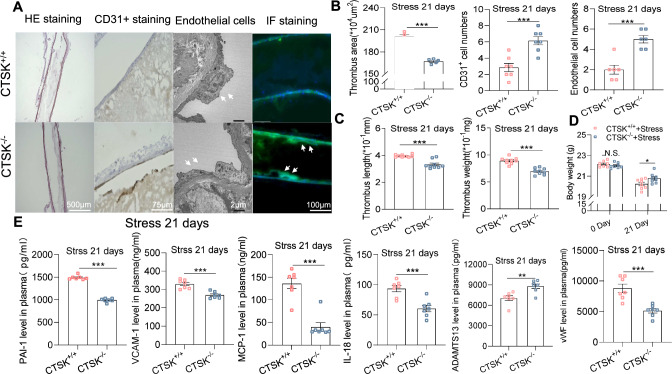
CTSK knockout lowered the artery endothelial damage and FeCl_3_-induced thrombosis in the stressed mice. **A**, **B** Representative images and quantitative data depicting the thrombus area [hematoxylin and eosin (H&E) staining: *left panels*] and the numbers of CD31^+^ cells (immunostaining: *left middle panels*) and endothelial cells of the thrombotic lesions (TEM: *right middle panels*) in the two groups (*n* = 5–7). The representative immunofluorescence shows CD31^+^ cells in both experimental groups (*right panels*). **C** The quantitative data of the weight and length of the thrombi in CTSK^+*/*+^ and CTSK^*−/−*^ mice after 2 weeks of immobilized stress (*n* = 9). **D** Body weights of the two groups on days 0 and 21 (*n* = 8). **E** ELISA results for the levels of blood PAI-1, MCP-1, VCAM-1, IL-18, ADAMTS13, and vWF on day 21 of the stress (*n* = 7). Results are mean ± SEM. *p < 0.05, **p < 0.01, ***p < 0.001, N.S. (not significant) vs. the stressed CTSS^+*/*+^ group by one-way ANOVA and Tukey’s post hoc test or Student’s *t-*test. *Scale bars* 2 μm for the TEM and 75, 100 and 500 μm for the others

**Fig. 3:**
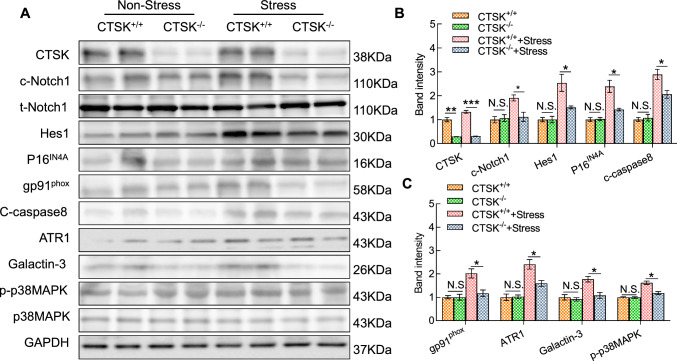
CTSK knockout improved the stress-related harmful alterations of the targeted protein levels in the FeCl_3_-treated arteries after 21 days of the stress protocol. **A**–**C** Total protein was isolated from the thrombotic arterial tissues of the stressed CTSK^+*/*+^ and stressed CTSK^*−/−*^ mice and applied to western blotting. Representative images and quantitative data show the levels of CTSK, c-Notch1, Hes1, p16^IN4A^, gp91phox, c-caspase8, ATR1, and p-p38MAPK proteins in both groups. Results are mean ± SEM (*n* = 3). *p < 0.05, **p < 0.01, ***p < 0.001 vs. corresponding non-stressed or stressed CTSS^+*/*+^ mice by one-way ANOVA and Tukey’s post hoc test

As shown in Fig. 2A, B, markedly less thrombus formation and significantly less endothelial cell loss were observed in the stressed arteries of the stressed CTSK^−/−^ mice on day 21 compared to the stressed CTSK^+/+^ mice. The representative immunofluorescence shows CD31^+^ cells in both experimental groups (Fig. 2, right panels). Similarly, compared to the control mice, there were dramatic reductions in thrombi size and weights in the stressed CTSK^−/−^ mice (Fig. 2C). The body weights were higher in the stressed CTSK^−/−^ mice on day 21 compared to the stressed CTSK^+/+^ mice (Fig. 2D). We also observed marked and significant improvements in the levels of plasma PAI-1, VCAM-1, MCP-1, IL-18, and ADAMST13 in the CTSK^−/−^ mice (Fig. 2E). As anticipated, CTSK deletion rectified the harmful alterations in the levels of c-Notch1, Hes1, p16^IN4A^, gp91^phox^, c-caspase8, ATR1, galatin-3, and p-p38MAPK proteins in the injured arterial tissues in response to stress (Fig. 3). Moreover, the quantitative PCR results revealed that CTSK deletion exerted beneficial effects on the changes of arterial proteolytic enzyme genes (CTSS, CTSL, MMP-2, and MMP-9), inflammation-related genes (ICAM-1, TNF-α, MCP-1, and TLR-4), and oxidative stress-related genes (gp91^phox^ and p22^phox^) (Suppl. Fig. S3). The CTSK deletion-mediated vasculoprotective effects might thus have been due to the reductions of oxidative stress, inflammation, and Notch1/Hes1-caspase8-dependent endothelial cell apoptosis in the arterial tissues of the mice subjected to our experimental conditions.

### CTSK inhibition mimicked the CTSK^−/−^-mediated vascular protection

**Fig. 4:**
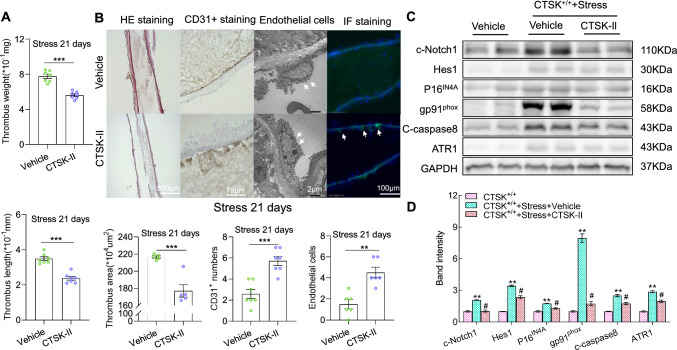
A CTSK inhibitor (CTSK-II) mitigated the stress-related arterial endothelial injury and thrombosis in CTSK^+*/*+^ mice in response to FeCl_3_. **A** Quantitative data of the length and weight of the thrombi (*n* = 7 each) in stressed CTSK^+*/*+^ mice loaded with the vehicle or CTSK-II (*n* = 7). **B** Representative images and combined quantitative data presenting the thrombus area (H&E staining: *left panels*) and the numbers of CD31^+^ cells (immunostaining: *left middle panels*) and endothelial cells of the thrombotic lesions (TEM: *right middle panels*) in the two groups (*n* = 5–7). Representative immunofluorescence shows CD31^+^ cells in both experimental groups (*right panels*). **C**, **D** Representative images and combined quantitative data of the levels of CTSK, c-Notch1, Hes1, p16^IN4A^, gp91phox, c-caspase8, and ATR1 proteins (*n* = 3). Results are mean ± SEM. *p < 0.05, **p < 0.01, ***p < 0.001 vs. non-stressed CTSS^+*/*+^ controls, ^#^*p* < 0.05 vs. stressed CTSS^+*/*+^ alone mice by unpaired Student’s *t*-test or one-way ANOVA and Tukey’s post hoc test. *Scale bars* 2 μm for the TEM and 75, 100 and 500 μm for the others

Similar to the results of the CTSK genetic modification, we observed that CTSK inhibition with its specific inhibitor resulted in not only smaller thrombus size and lower thrombus weights but also smaller thrombus areas in the injured vessel tissues of the stressed CTSK-II mice compared to the stressed control CTSK^+/+^ mice (Fig. 4A). CTSK-II loading prevented the carotid arteries’ endothelial cell loss (Fig. 4B). The quantitative data from the immunoblotting assays revealed that the inhibition of CTSK significantly lowered the levels of apoptotic proteins (c-Notch1, Hes1, p16^IN4A^, gp9^phox^, and c-caspase8) (Fig. 4C, D), suggesting that pharmacological interventions targeted toward CTSK can yield the same results as CTSK genetic intervention.

### CTSK silencing protected against the oxidative stress- and stress serum-induced apoptosis of HUVECs

**Fig. 5:**
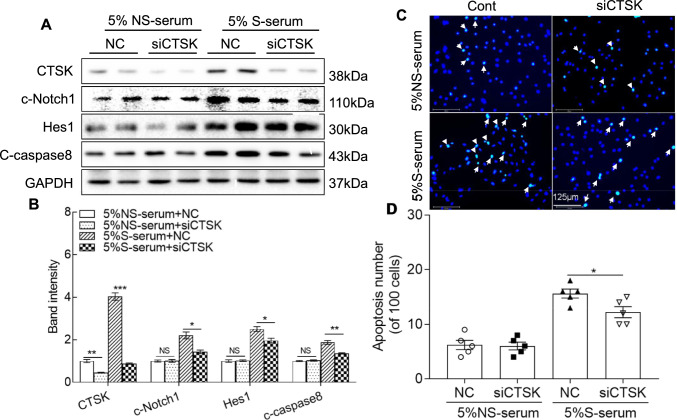
CTSK silencing (siCTSK) ameliorated the stressed serum (S-serum)-induced apoptosis-related protein expression in HUVECs. Following transfection with siRNA against CTSK (siCTSK) or a negative control (NC) for 48 h, the cells were incubated with 5% stressed serum (from 10-day-stressed mice) or 5% non-stressed serum (NS-serum) for 24 h and then subjected to the apoptosis and western blotting assays. **A**, **B** Representative western blotting images and combined quantitative data presenting the investigated protein levels (CTSK, c-Notch1, Hes1, and c-caspase8) in the four groups (*n* = 4 each). **C**, **D** Representative TUNEL images and combined quantitative data of the numbers of TUNEL-positive cells induced by both serum stimulations in HUVECs (*n* = 5). Data are mean ± SEM, *p < 0.05, **p < 0.01, ***p < 0.001 vs. the corresponding controls by one-way ANOVA, followed by Tukey’s post hoc tests. *Scale bars* 30, 125 μm

**Fig. 6:**
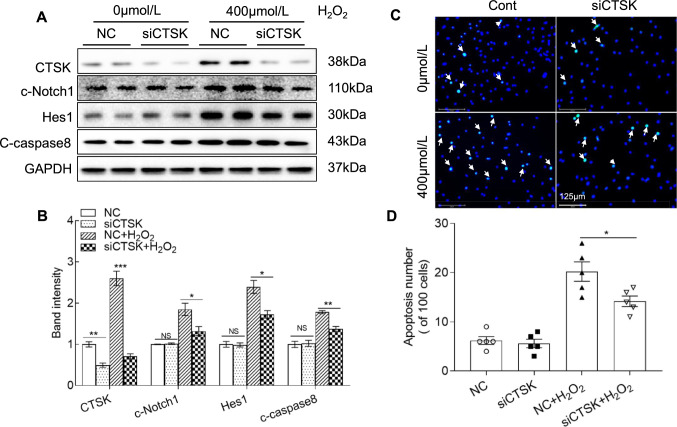
CTSS silencing (siCTSK) ameliorated oxidative stress-induced apoptosis-related protein expression in HUVECs. Following transfection with siRNA against CTSK (siCTSK) or a negative control (NC) for 48 h, the cells were incubated with H_2_O_2_ at the indicated concentrations (0 or 400 μmol/L) for 24 h and then subjected to the apoptosis and western blotting assays. **A**, **B** Representative immunoblot images and combined quantitative data showing the levels of CTSK, c-Notch1, Hes1, and c-caspase8 in HUVECs (*n* = 4). **C**, **D** Representative TUNEL-staining images and combined quantitative data of the apoptosis in the four groups (*n* = 5 each). Data are mean ± SEM. *p < 0.05, **p < 0.01, ***p < 0.001 vs. corresponding controls by one-way ANOVA and Tukey’s post hoc tests. *Scale bar* 125 μm

Next, to explore the beneficial CTSK silencing-mediated effect on endothelial cell apoptosis and its mechanism, we cultured HUVECs in the presence of H_2_O_2_ (0, 200, and 400 μM) for 24 h and then subjected the cells to the biological assay and TUNEL staining. The quantitative PCR and western blotting results demonstrated that the expression of CTSK protein was sensitive to H_2_O_2_ induction (Suppl. Fig. S4A, B). The H_2_O_2_ treatment also exerted dose-dependent effects on the changes of c-Notch1, Hes1, and c-caspase8 protein levels and HUVEC apoptosis (Suppl. Fig. S4C,D). As anticipated, CTSK knockdown by siCTSK lowered the CTSK protein expression (Figs. 5A, B, 6A, B). We observed that the levels of c-Notch1, Hes1, and c-caspase8 proteins were elevated in the lysates of the HUVECs treated with oxidative stress or 5% stress serum, and these alterations were rectified by CTSK knockdown. The TUNEL staining demonstrated that CatK deletion mitigated the 5% stress serum- and H_2_O_2_- induced HUVEC apoptosis (Figs. 5C, D, 6C, D), suggesting that CTSK knockdown exerted a protective action against HUVEC apoptosis in response to oxidative stress and stressed serum.

### CTSK overexpression accelerated the oxidative stress- and stress serum-induced apoptosis of HUVECs

**Fig. 7:**
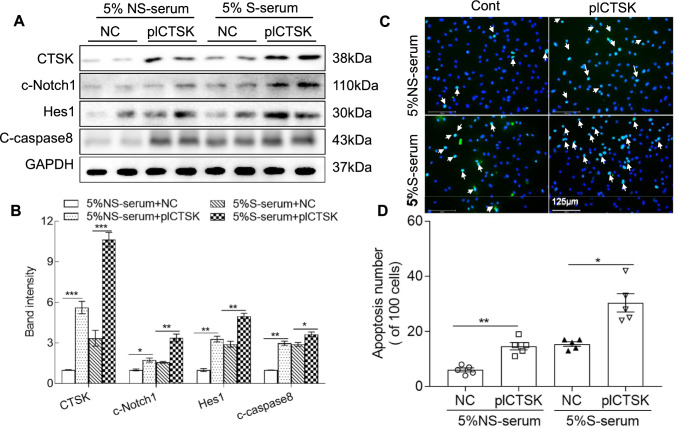
Plasmid-CTSK (pl-CTSK)-mediated CTSK overexpression enhanced the stressed serum (S-serum)-induced cell apoptosis. Following transfection with pl-CTSK (pcDNA3.1( +)-CTSK plasmid) or empty vector (NC) for 48 h, the cells were incubated with 5% stressed serum (from 10-day-stressed mice) or 5% non-stressed serum (NS-serum) for 24 h and then subjected to the apoptosis and western blotting assays. **A**, **B** Representative western blotting images and combined quantitative data showing the investigated protein levels (CTSK, c-Notch1, Hes1, and c-caspase8) in the four groups (*n* = 4 each). **C**, **D** Representative TUNEL images and combined quantitative data showing TUNEL-positive apoptotic cells induced by both serum stimulations (*n* = 5). Data are mean ± SEM, *p < 0.05, **p < 0.01, ***p < 0.001 vs. the corresponding controls by one-way ANOVA and Tukey’s post hoc tests. *Scale bar* 125 μm

**Fig. 8:**
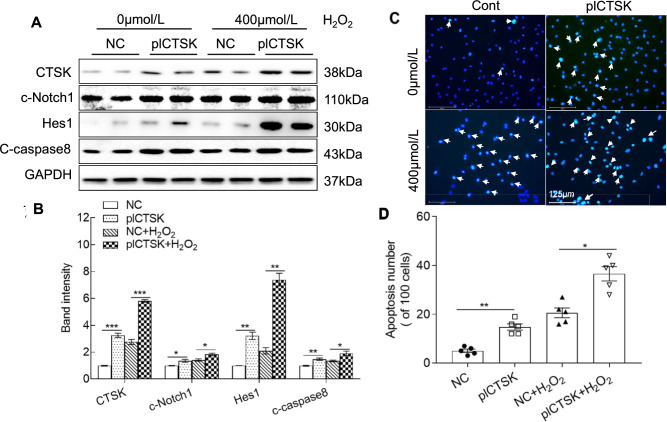
Plasmid-CTSK (pl-CTSK)-mediated CTSS overexpression enhanced the oxidative stress-induced cell apoptosis. Following transfection with pl-CTSK (pcDNA3.1(+)-CTSK plasmid) or empty vector (NC) for 48 h, the cells were treated with H_2_O_2_ at 0 or 400 μmol/L for 24 h and then subjected to apoptosis and western blotting assays. **A**, **B** Representative images and combined quantitative data showing the investigated protein levels (CTSK, c-Notch1, Hes1, and c-caspase8) in HUVECS in response to oxidative stress (*n* = 4). **C**, **D** Representative TUNEL staining images and combined quantitative data showing the apoptosis in HUVECs in response to H_2_O_2_ stimulation. Data are mean ± SEM (*n* = 5–6). *p < 0.05, **p < 0.01, ***p < 0.001 vs. the corresponding controls by one-way ANOVA and Tukey’s post hoc tests. *Scale bar* 125 μm

To further examine the consequences of CTSK overexpression, we analyzed cell lysates for the levels of the above-mentioned molecules. The results indicated that CTSK overexpression increased stress serum- and oxidative stress-induced CTSK, c-Notch1, Hes1, and c-caspase8 (Figs. 7A, B, 8A, B). The oxidative stress and 5% stress serum accelerated HUVEC apoptosis (Figs. 7C, D, 8C, D). Together, the in vivo and in vitro results of the genetic CTSK modifications thus provide a mechanistic explanation of CTSK’s participation in endothelial cell damage and apoptosis under stress conditions.

### CTSK participated in c-Notch1 processing in endothelial cells

**Fig. 9:**
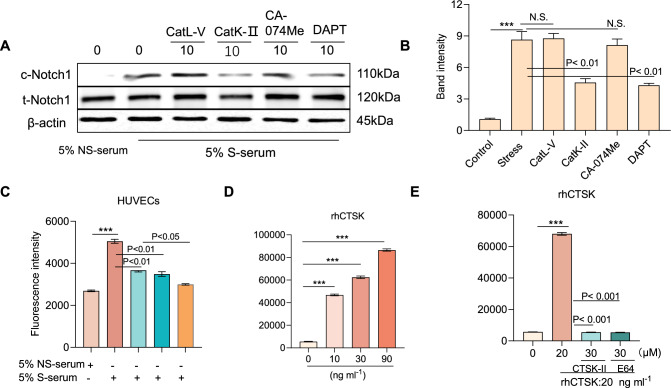
Pharmacological CTSK suppressed c-Notch1 processing in HUVECs in response to stress serum. **A**, **B** Cells were treated with stress serum for 24 h in the presence or absence of various protease inhibitors including a specific CTSK (CTSK-II), CTSL (CTSL-V), CTSB (CA-074Me), and γ-secretase (DAPT) at the indicated concentrations. The lysates were separately immunoblotted with c-Notch1, t-Notch1, and β-actin antibodies. The representative images (**A**) and combined quantitative data (**B**) show the investigated protein levels. **C** HUVECs were cultured with 5% stress serum for 24 h at 37 °C; the lysate (total protein 200 μg) was incubated with 8 μmol/L of an intramolecularly quenched fluorogenic peptide probe in the presence or absence of CTSK-II, L-685, or CTSK-II + L-685, and the CTSK activity was then assayed using a plate reader. **D**, **E** Recombinant human CTSK (rhCTSK) was reacted with the fluorogenic peptide probe for 24 h (pH 6.8) in the presence or absence (**D**) of a Cat inhibitor (**E**: CTSK-II, E64) at the indicated concentrations, followed by a fluorescence assay. Data are mean ± SEM of six independent experiments performed in triplicate. *p < 0.01, ***p < 0.001 vs. corresponding controls by ANOVA and Tukey’s post hoc tests

A presenilin/γ-secretase-independent mechanism has been shown to play an important role in Notch1 signaling transduction. If there are proteases responsible for Notch1 cleavage, we should be able to detect the reduction of cleaved Notch1 (c-Notch1) by treatment with related specific protease inhibitors. To test this hypothesis, we exposed HUVECs to stress serum in the presence or absence of various protease inhibitors (i.e., DAPT, CTSK-II, CA-07Me, and CTSL-V) at various concentrations. We used a γ-secretase inhibitor (DAPT) as a positive control in in vitro experiments. Similar to DAPT, a specific CTSK inhibitor (CTSK-II) and a nonspecific cathepsin inhibitor (E64*d*) each markedly lowered the levels of c-Notch1 protein in HUVECs; however, neither an MMP inhibitor (GM6001) nor other cathepsin-specific inhibitors (including the CTSL inhibitor CTSL-V and the CTSB inhibitor CA-07Me) affected the c-Notch1 protein levels (Fig. 9A). Thus, among the members of the cathepsin family, CTSK appears to participate in Notch1 cleavage in HUVECs under stress conditions.

**Fig. 10:**
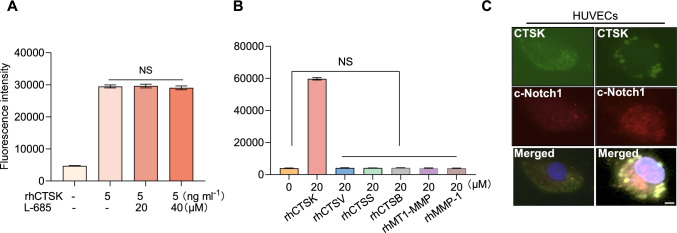
CTSK-mediated γ-secretase substrate degradation. **A** Recombinant human CTSK (rhCTSK) was incubated with a fluorogenic peptide probe for 24 h (pH 6.8) in the presence or absence of a γ-secretase inhibitor (L-685) at the indicated concentrations, followed by a fluorescence assay. **B** Four recombinant cathepsins (rhCTB, rhCTSL, rhCTSK, and rhCTSS) and rhMMPs were incubated with a fluorogenic probe for 24 h (pH 6.8 for CatK; pH 7.4 for MMP) and then assayed for fluorescence. Data are mean ± SEM of six independent experiments performed in triplicate. N.S. vs. corresponding controls by ANOVA and Tukey’s post hoc tests. **C** Representative immunofluorescence images show CTSK in the intramembranous regions and the accumulation of c-Notch1 in the nuclei. *Scale bars* 50 μm

To further investigate whether member of the cathepsin family contribute to Notch1 activation, we conducted a series of γ-secretase activity assays with the recombinant human cathepsins (including rhCTSK, rhCTSS, rhCTSL, and rhCTSL) and the cell lysates in the presence of several specific cathepsin inhibitors. As anticipated, similar to the case with the inhibition of γ-secretase, CTSK inhibition lowered the HUVEC-lysate-associated substrate degradation and exerted an additive effect by combination with γ-secretase inhibition (Fig. 9C). The γ-secretase substrate was sensitive to rhCTSK in a dose-dependent manner, and this activity was dramatically suppressed by CTSK-II and E64 but was not suppressed at all by the γ-secretase inhibitor L-685,458 (L-685, Fig. 10A) at the concentrations used. A parallel analysis of other cathepsin family members (rhCTSB, rhCTSV, and rhCTSS) and MMP family members (rhMT1-MMP and rhMMP-1) revealed no effect (Fig. 10B). Immunofluorescence showed slight staining signals for CTSK and the Notch1 intracellular domain localized in the intramembranous regions of untreated HUVECs (Fig. 10C). Interestingly, exposure to stress serum facilitated the accumulation of c-Notch1 in the nuclei. These results thus further supported our hypothesis that CTSK participates in the intramembranous cleavage of Notch1 in vascular endothelial cells.

## Discussion

Arterial endothelial cell injury is a trigger for thrombotic cardiovascular events in CCVD patients with and without chronic psychological stress (CPS) [29–31]. The identification of novel molecular targets that can be used to prevent arterial endothelial injury/apoptosis will contribute to therapeutic strategies to preempt thrombosis in atherosclerotic CCVD patients with CPS. The important non-traditional and traditional roles of lysosomal cysteinyl cathepsins in various pathological conditions have been confirmed by clinical and experimental investigations [23–27]. Although those studies uncovered proteolysis-dependent and -independent mechanisms underlying atherosclerotic CCVD and vascular regeneration, the limited number of more recent studies have described only the increased expressions of CTSK and CTSS mRNAs and proteins in cardiovascular tissues injured by stress [32, 33].

Here we investigated the beneficial effects of genetic and pharmacological inhibitions targeting CTSK in stress-related arterial thrombosis in mice in response to FeCl_3_. The most important finding of this study is that CTSK deficiency was resistant to stress/FeCl_3_-induced endothelial injury/loss and thrombus formation. At the cellular and molecular levels, CTSK deficiency was observed to prevent: (i) the harmful changes in plasma PAI-1, vWF, and ADAMTS; (ii) oxidative stress-related NADPH oxidase (gp91^phox^ and p22^phox^)-related and senescence-related molecule (p16^INK4A^ and ATR1) activation; (iii) inflammatory TNF-α/TRL4-galactin-3 signaling activation; and (iv) Notch1/Hes1-caspase-8 activation-mediated endothelial apoptosis. The CTSK inhibitor also exerted a vascular benefit on FeCl_3_-induced thrombus formation in mice under our experimental conditions. In vitro, the CTSK overexpression and knockdown respectively enhanced and lowered the levels of Notch1, Hes1, and c-caspase-8 and cellular apoptotic events in HUVECs in response to oxidative stress and stress serum, providing evidence and a mechanistic explanation of the involvement of CTSK in Notch1/Hes1-c-caspase-8 apoptotic signaling activation in stressed arterial endothelial apoptosis and thrombosis.

CTSK has been shown to be overexpressed in stressed muscle, adipose, kidney, and cardiovascular tissues [24, 33–36]. Laboratory studies revealed that inflammatory cytokines and oxidative stress stimulate CTSK expression and activity in cardiovascular and renal cells under various pathological conditions [24, 32]. In the present study’s cultured HUVECs, the CTSS gene and protein expressions were sensitive to H_2_O_2_ and stress-serum induction. Taken together with the data of the western blotting and PCR assays revealing that the thrombotic lesions had elevated CTSK protein, these results indicate that there may be a close relationship between the carotid artery’s CTSK expression and thrombus formation in mice in response to stress/FeCl_3_. There have been limited studies of the roles of cathepsins and their endogenous inhibitors (stefin A) in the thrombosis of atherogenic-complicated lesions [37]. Among the members of the cathepsin family, CTSS has been proposed to play a pivotal role in thrombosis [14]. Both in vitro and in vivo, the inhibition of cathepsins and the overexpression of stefin A have been observed to exert an anti-thrombosis effect [38]. Of importance, our present findings demonstrated that stress augments the FeCl_3_-induced formation of thrombi, and this effect was rectified by CTSK knockout and its inhibitor. The ability of chronic stress to elevate the expression of CTSK is thus likely to have been involved in the acceleration of the arterial thrombosis in mice under the stress conditions used herein.

Notch signaling has been shown to control cell fates and signal integration in development [39]. Laboratory evidence indicates that among the Notch receptors, Notch1/Hes1 signaling modulates not only cell proliferation but also apoptosis in the initiation and progression of tumors and cardiovascular diseases [26, 40]. One of our earlier investigations demonstrated that CTSK deficiency decreased the levels of the ischemic muscle c-Notch1 protein [26]. Our present results demonstrate that among the members of the cathepsin family, a pharmacological inhibition of only CTSK lowered c-Notch1 levels in HUVECs under stress-serum conditions; this was not achieved by other cathepsins (CTSS, CTSL, and CTSB) or MMPs (MT1-MMP and MMP-1). Consistently, the γ-secretase substrate was sensitive only to rhCTSK in a dose-dependent manner, and this cleaving activity was completely suppressed by its specific inhibitor CTSK-II. Thus, these findings suggest that CTSK could participate in in the proteolytic activation of Notch1, although there was no direct evidence.

We also demonstrated that CTSK-mediated caspase-8 activation is a key step in vascular smooth muscle cell apoptosis induced by oxidative stress in vitro and in vivo [23]. One of our significant present findings is that stress resulted in a harmful change in the levels of CTSK, c-Notch1, Hes1, c-caspase8, and p-p38MPAK, and these effects were rectified by CTSK deletion. Consistently, the quantitative data of the CD31 immunostaining and transmission electron microscopy revealed that the numbers of endothelial cells were lower in the stressed arterial tissues compared to the control non-stressed arterial tissue; these changes were also rectified even in the stressed CTSK^−/−^ mice. The pharmacological CTSK inhibition yielded the same results in stressed CTSK^+/+^ mice. In HUVECs, the knockdown and the overexpression of CTSK respectively decreased and enhanced c-Notch1 levels and its downstream signaling, providing the first evidence and mechanistic explanation of how CTSK modulates endothelial apoptosis and thrombus formation in mice under our stress conditions.

Clinical and laboratory evidence has suggested that oxidative stress plays an important role in the senescence and apoptosis of endothelial cells and in the secretion of cytokines and chemokines, leading to thrombus formation and its related cardiocerebrovascular events in patients with CCVDs [41, 42]. Our present findings revealed that the stressed arterial tissues had elevated levels of p22^Phox^ and gp91^Phox^ genes and/or proteins. The genes p22^Phox^ and gp91^Phox^ are important membrane subunits of NADPH oxidase [43]. NADPH oxidase is one of the major sources of reactive oxygen species, and it has been documented that pharmacological and genetic suppressions of NADPH oxidase components mitigated vascular endothelial cells’ senescence and dysfunction, leading to the prevention of endothelial cell apoptosis and atherosclerotic lesion formation in several animal models [34, 43, 44]. The mice that were subjected to immobilized stress in the present study showed accelerated FeCl_3_-induced thrombus formation, and we therefore propose that the elevation of stress-induced oxidative stress might contribute to vascular senescence and apoptosis in mice that have been subjected to combined stress and FeCl_3_ injury. Our results also demonstrated that genetic and pharmacological interventions of CTSK protected against harmful alterations (c-Notch1, Hes1, p16^IN4A^, gp91^phox^, c-caspase8, ATR1, galatin-3, and p-p38MAPK) and thrombosis in the arteries of mice, accompanied by reductions of the gp91^Phox^ and p22^Phox^ expressions in the arterial injured tissues of the stressed mice. These findings indicate that the beneficial vascular effects of pharmacological and genetic CTSK interventions occur, at least partially, through the modulation of NADPH oxidase-mediated oxidative stress production in mice under the present experimental conditions.

Accumulating evidence indicates that CPS produces an inflammatory overaction in several tissues (e.g., ischemic muscle and adipose tissues) [4, 35]. Our previous findings revealed that compared to non-stressed mice, stress accelerated a high-fat-diet-induced inflammation response in atherosclerotic plaques [25, 45]. The stressed arterial tissues had enhanced inflammation-related gene and/or protein expressions (i.e., TNF-α, TLR-4, ICAM-1, ATR1, galactin-3, and MMP-2/-9) as well as enhanced intracellular signal protein levels (c-Notch1, Hes1, p16^IN4A^, c-caspase8, and p-p38MAPK); these changes were rectified by CTSK inhibition. Pro-inflammatory influences of these molecules on the process of vascular endothelial cell senescence and apoptosis, the thrombotic state, and atherosclerotic lesion initiation and progression have been sufficiently investigated by clinical and laboratory studies by our and other groups [34, 35, 45, 46]. We thus speculate that the chronic stress in the present study accelerated the development of FeCl_3_-induced vascular harmful alteration by increasing inflammatory actions. Our present results revealed that CTSK inhibition rectified the stressed arterial inflammation in response to stress/FeCl_3_ injuries. It has also been demonstrated that CTSK deletion prevented macrophage infiltration and vascular remodeling [32]. Galactin-3 has been shown to be closely linked to inflammatory cell activation in humans and animals [47, 48]. Collectively, these findings suggest the ability of CTSK inhibition to ameliorate an inflammatory status, and they indicate that the reduction of inflammatory cytokines associated with the inactivation of TNF-α/TLR-4 and ATR1/galactin-3 signaling can exert a salutary effect on vascular endothelial cell injury and apoptosis, thereby improving thrombus formation under stress conditions.

Members of the ADAMTS family have shown important roles in cell adhesion and signaling. Among these family members, ADAMTS13 degrades unusually large vWF multimers into small vWF fragments, and this product lowers vWF coagulation abilities [49]. Experimental and clinical studies indicated that the imbalance of vWF and ADAMTS13 is closely linked to thrombotic events in CCVD patients under pathological conditions [50]. Vascular endothelial cells produce mainly unusually large fragments of vWF and ADAMTS13 [50, 51]. Our present in vivo results revealed that the CTSK inhibitor lessened the stress/FeCl_3_-induced arterial endothelial apoptosis, accompanied by a reduction of increased NADPH oxidase components. Our in vitro CTSK genetic modification yielded the same results in HUVECs. Oxidative stress has been shown to produce a prothrombotic state via the regulation of ADAMTS13 and vWF expressions and/or modifications [52]. These findings thus suggest that both genetic and pharmacological treatments might rectify the changes in arterial oxidative stress, resulting in a vasculoprotective effect on endothelial damage/apoptosis and the imbalance of endothelial cell-derived vWF and ADAMTS13, thereby preventing thrombosis in mice subjected to our stress conditions. It should be noted that the 3 weeks of restraint stress elevated the plasma PAI-1 level in the mice, and this alteration was rectified by the genetic and pharmacological interventions of CTSK, indicating that the vasculoprotective effects of CTSK inhibition are likely also attributable, at least in part, to an attenuation of the PAI-1 elevation in the mice that had been subjected to the stress/FeCl_3_.

There are several study limitations to address. First, the chronic variable stress model used herein is an animal stress model that cannot completely mimic human psychological stress. The study could not clarify the CTSK-mediated cleavage site of Notch1 in vivo or in vitro. Thus, it should be stated that while the expression of Notch signaling molecules were affected by CTSK and stress manipulations there is no direct evidence to support Notch signaling was responsible for CTSK mediated effects both in vivo and in vitro. It also remains unclear whether both ADAMTS13 and PAI-1 gene expressions and protein expressions directly depend on CTSK activity, and we were also unable to identify a mediator that links CTSK activity and ADAMTS13 expression in endothelial cells and mice. lastly, we could not clarify the role of CTSK in vein thrombus formation in mice under our experimental conditions. Further research is necessary to investigate these issues.

In summary, the expressions of cysteinyl CTSK gene and protein were elevated in the FeCl_3_-treated arteries of mice under our experimental conditions. The immobilization stress resulted in FeCl3-treated artery endothelial cell apoptosis and thrombosis associated with the injured artery inflammation, oxidative stress production, proteolysis, and imbalance between plasma vWF and ADAMTS13, and these alterations were reversed by both the genetic and pharmacological CTSK inhibitions. This study appears to be the first to show the protective action of CTSK inhibition regarding arterial endothelial cell apoptosis and thrombus formation, indicating that a selective synthetic CTSK inhibitor may have potential utility in the treatment of thrombotic events in CCVD patients who have experienced chronic psychological stress.

### Supplementary Information

Below is the link to the electronic supplementary material.

Supplementary file1 (PDF 1204 KB)

## Data Availability

Not applicable.
