# Supplementary material for: Cathepsin K deficiency prevented stress-related thrombosis in a mouse FeCl3 model
Source: Cell Mol Life Sci. 2024 May 4;81(1):205. doi: 10.1007/s00018-024-05240-0 (PMC11069486; doi:10.1007/s00018-024-05240-0)
Supplement: Supplementary file 1 — Supplementary file1 (PDF 1204 KB) [file 18_2024_5240_MOESM1_ESM.pdf]

SUPPLEMENTAL MATERIALS

Cathepsin K Deficiency Prevented Stress-Related Thrombosis in in a Mouse $FeCl_3$ Model

Xueying Jin, MD; Xueling Yue, PhD; Zhe Huang, MD, PhD; Xiangkun Meng, MD; Shengnan Xu, MD, PhD; Yuna Wu, MD; Ying Wan, MD, PhD; Aiko Inoue, PhD; Megumi Narisawa, MD; Lina Hu, MD, PhD; Guo-Ping Shi, DSc; Hiroyuki Umegaki, MD, PhD; Toyoaki Murohara, MD, PhD; Yanna Lei, MD, PhD; Masafumi Kuzuya, MD, PhD; Xian Wu Cheng, MD, PhD, FAHA

***Corresponding author:**

Prof. Xian Wu Cheng, PhD Xueling Yue or Dr Yanna Lei, Department of Cardiology and Hypertension, Jilin Provincial Key Laboratory of Stress and Cardiovascular Disease, Yanbian University Hospital, 1327 Juzijie, Yanji 133000, China. Email: chengxw0908@163.com, 1945199153@qq.com, or leiyannalyn@126.com

Supplemental Materials

Supplemental Table S1-2

Figure S1-4

Suppl. Table S1: Mouse stress protocol.

Day		Stress	Procedure
Day 1	Cage tilt	4 hour	Overnight illumination
Day 2	Cage horizontal	4 hour	Meantime damp environmen
Day 3	Cage tilt	4 hour	Overnight illumination
Day 4	Cage horizontal	4 hour	Meantime damp environmen
Day 5	Cage tilt	4 hour	Overnight illumination
Day 6	Cage horizontal	4 hour	Meantime damp environmen
Day 7	Cage horizontal	4 hour	Rest

The mouse received several different kinds of stressors from Sunday to Saturday and we changed the stress order randomly.

Suppl. Table S2. Primer sequences for mice used for quantitative real-time PCR

Gene	Forward primer	Reverse primer
p22phox	AACTACCTGGAGCCAGTTGAG	AATTAGGAGGTGGTGGAATATCGG
gp91phox	ACTTTCCATAAGATGGTAGCTTGG	GCATTCACACACCACTCAACG
MMP-2	CCCCATGAAGCCTTGTTTACC	TTGTAGGAGGTGCCCTGGAA
MMP-9	CCAGACGCTCTTCGA GAACC	GTTATAGAAGTGGCGGTTGT
AT1a	TTTCCAGATCAAGTGCATTTTGA	AGAGTTAAGGGCCATTTTGCTTT
ICAM-1	CCCCGCAGGTCCAATTC	CCAGAGCGGCAGAGCAA
VCAM-1	ACAAAACGATCGCTCAAATCG	GGTGACTCGCAGCCCGTA
MCP-1	GCCCCACTCACCTGCTGCTACT	CCTGCTGCTGGTGATCCTCTTGT
TLR 4	AGTGGGTCAAGGAACAGAAGCA	CTTTACCAGCTCATTTCTCACC
TNF-α	AGGCTGCCCCGACTACGT	GACTTTCTCCTGGTATGAGATAGCAAA
CTSL	GGCAACCCGATGCGC	TGTGTGACTCCTGTGAAGAACCA
CTSS	GTGGCCACTA AAGGGCCTG	ACCGCTTTTGTAGAAGAAGAAGGAG
CTSK	AGCAGGCTGGAGGACTAAGGT	TTTGTGCATCTCAGTGGAAGACT
GAPDH	ATGTGTCCGTCGTGGATCTGA	ATGCCTGCTTCACCACCTTCT

AT1α: angiotensin receptor 1alpha, CatS: cathepsin S, CatL: cathepsin L, CXCR4: C-X-C motif chemokine receptor-4, GAPDH: gluceradehyde-3-phosphate dehydrogenase, IL-1β: interleukin-1beta, ICAM-1: intercellular adhesion molecule-1, MCP-1: monocyte chemoattractant protein-1, MMP-2: matrix metalloproteinase-2, MMP-9: matrix metalloproteinase-9, NOX1: NADPH oxidase 1TLR-2: toll-like receptor-2, , SDF-1α: stromal cell-derived factor-1alpha, VCAM-1: vascular cell adhesion molecule-1, TNF-α: tumor-necrosis factor-alpha.

Supplementary Figure Legends

Suppl. Fig. S1. The 3-week stress lowered the inguinal adipose volumes, body weight, and expression of the investigated genes in the arterial tissues of the CTSS$^{+/+}$ mice that underwent the stress/$FeCl_3$ protocols. **A,B:** Representative photo and quantitative data showing the weights of the inguinal adipose tissue (n=9). **C**: The changes in body weight during the 14-day follow-up in both groups (n=8). **D,E:** Results of the quantitative immunohistological and H&E staining analyses for CD31$^+$ cell numbers and thrombus area. **F–I:** Results of the PCR for the proteolytic enzyme genes (CTSK, CTSL, CTSL, MMP-2, and MMP-9), inflammation-related genes (ICAM-1, TNF-α, MCP-1, and TLR-4), and oxidative stress-related genes (gp91^{phox} and p22^{phox}) in thrombotic carotid artery tissue of the mice in the two experimental groups (n=5–6). Data are mean ± SEM. *p<0.05, **p<0.01, ***p<0.001, N.S. (not significant) vs. non-stress mice (Cont) by unpaired Student's *t*-test. Scale bar: 5 mm.

Suppl. Fig. S2. Stress produced a harmful change in the plasma VCAM-1, vWF, PAI-1, ADAMTS13, IL-18, and MCP-1 protein levels of the mice at day 21. **A–F:** VCAM-1, vWF, PAI-1, ADAMTS13, IL-10, and MCP-1 measured by ELISA in plasma of mice in the two groups (n=7 each). **p<0.01, ***p<0.001 vs. corresponding stressed CTSS$^{+/+}$ by one-way ANOVA, followed by Tukey's post hoc tests.

Suppl. Fig. S3. CTSK$^{-/-}$ reduced the expression of investigated genes in the arterial tissues of the stressed mice. **A–E:** PCR data of the levels of CTSL, CTSS, ICAM-1, TNF-α, gp91^{phox}, MCP-1, and p22^{phox} mRNAs. Data are mean ± SEM (n=5). *p<0.05, **p<0.01, ***p<0.001, N.S. vs. corresponding stressed CTSS$^{+/+}$ mice by one-way

ANOVA and Tukey's post hoc tests or unpaired Student's *t*-test.

Suppl. Fig. S4. Oxidative stress increased the CTSK gene expression and cell apoptosis. HUVECs were stimulated with various concentrations of H_2O_2 (0, 200, 400 μmol/L) for 24 hr. **A,B:** Representative TUNEL images and quantitative data showing the numbers of apoptotic cells treated with H_2O_2 at the indicated concentrations. **C,D:** HUVECs were treated with the indicated concentrations of H_2O_2 for 24 hr and then applied to western blotting. Representative western blotting images and quantitative analysis present the levels of CTSK, c-Notch1, Hes1, and C-caspase8 protein. Results are mean ± SEM (n= 4). *p<0.05, **p<0.01, ***p<0.001 vs. corresponding controls by one-way repeated measures ANOVA and Tukey's post hoc tests.

Figure S1

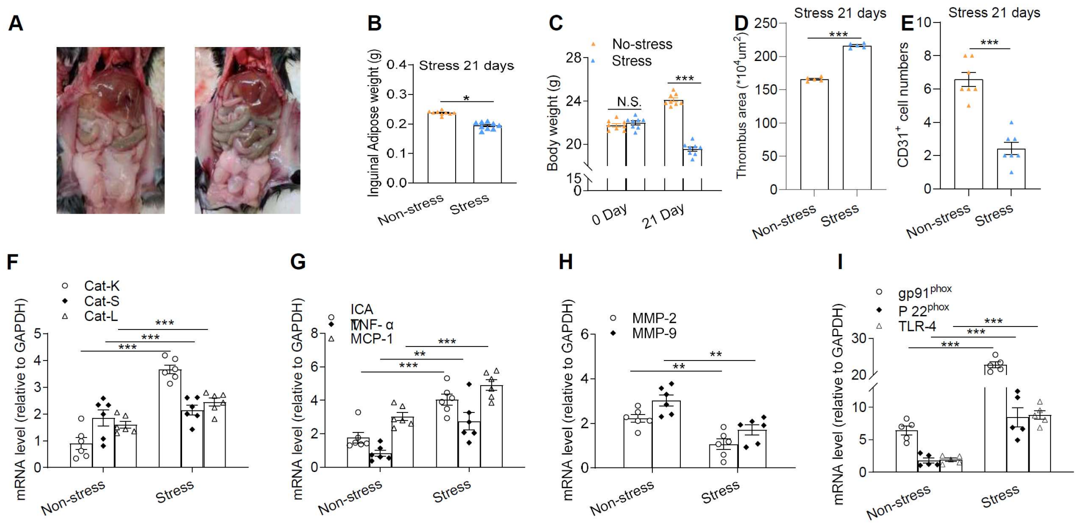
A
B
Stress 21 days
Inguinal Adipose weight (g)
Non-stress
Stress
C
No-stress
Stress
Body weight (g)
N.S.
0 Day
21 Day
D
Stress 21 days
Thrombus area (*10⁴um²)
Non-stress
Stress
E
Stress 21 days
CD31⁺ cell numbers
Non-stress
Stress
F
Cat-K
Cat-S
Cat-L
mRNA level (relative to GAPDH)
Non-stress
Stress
G
ICA
TNF- α
MCP-1
mRNA level (relative to GAPDH)
Non-stress
Stress
H
MMP-2
MMP-9
mRNA level (relative to GAPDH)
Non-stress
Stress
I
gp91phox
P 22phox
TLR-4
mRNA level (relative to GAPDH)
Non-stress
Stress

Figure S2

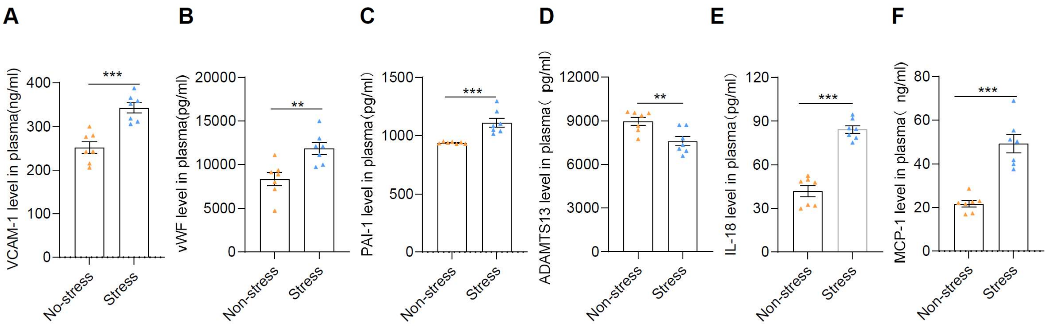
A
B
C
D
E
F
VCAM-1 level in plasma(ng/ml)
vWF level in plasma(pg/ml)
PAI-1 level in plasma(pg/ml)
ADAMTS13 level in plasma（ pg/ml)
IL-18 level in plasma (pg/ml)
MCP-1 level in plasma（ ng/ml)
No-stress
Non-stress
Stress
***
**

Figure S3

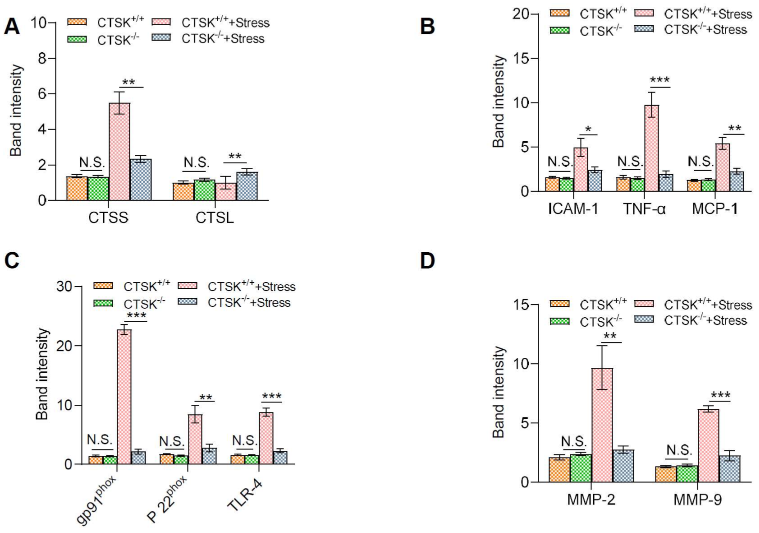
A
B
C
D
CTSK+/+
CTSK-/-
CTSK+/++Stress
CTSK-/-+Stress
Band intensity
N.S.
CTSS
CTSL
ICAM-1
TNF-α
MCP-1
gp91phox
P 22phox
TLR-4
MMP-2
MMP-9

Figure S4

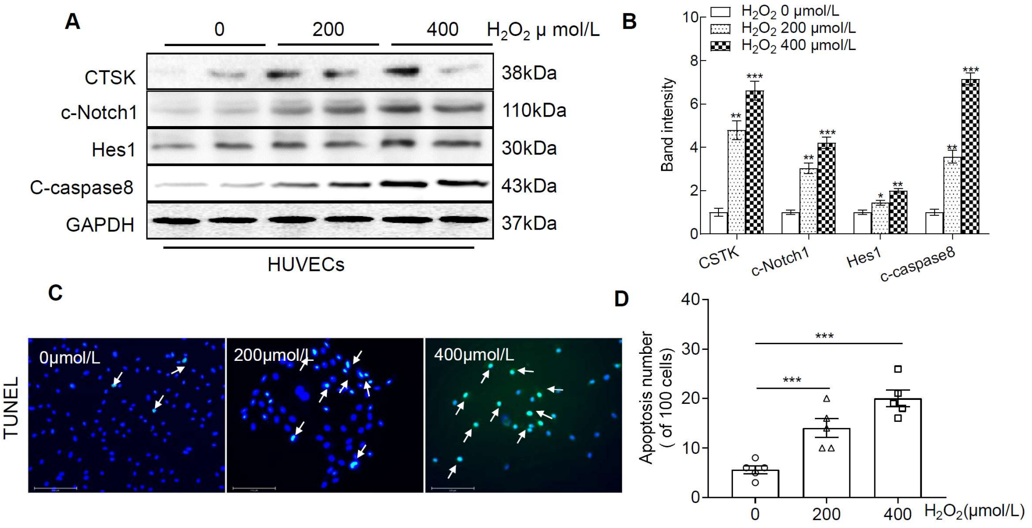

A
0
200
400
H_2O_2 μ mol/L
CTSK
c-Notch1
Hes1
C-caspase8
GAPDH
38kDa
110kDa
30kDa
43kDa
37kDa
HUVECs
B
H_2O_2 0 μmol/L
H_2O_2 200 μmol/L
H_2O_2 400 μmol/L
Band intensity
CSTK
c-Notch1
Hes1
c-caspase8
C
TUNEL
0μmol/L
200μmol/L
400μmol/L
D
Apoptosis number
(of 100 cells)
***
0
200
400
H_2O_2(μmol/L)
